# Wild Edible Mushrooms of Jharkhand: Nutrient-Dense Seasonal Foods to Improve Dietary Diversity among Indigenous Communities

**DOI:** 10.12944/CRNFSJ.13.1.4

**Published:** 2025-03-25

**Authors:** Archna Singh, Geetanjali Singh, Ridhima Kapoor, Ayushi Dhasmana, Suparna Ghosh-Jerath

**Affiliations:** 1 Department of Biochemistry, All India Institute of Medical Sciences, New Delhi, India; 2 Department of Botany, Dr Shyama Prasad Mukherjee University, Ranchi, Jharkhand, India; 3 The George Institute for Global Health INDIA, New Delhi, India

**Keywords:** Dietary Diversity, Indigenous Populations, Mushroom Consumption, Nutrient Composition, Wild Mushrooms

## Abstract

Wild edible mushrooms can contribute to dietary diversity, especially among undernourished indigenous communities. A cross-sectional mixed-methods study was conducted to document the availability, taxonomic classification, nutritive values, and consumption frequency of wild mushrooms in four indigenous communities of Jharkhand. Over 70 wild mushrooms were reported during free listing with 35 being regularly consumed during the monsoon season, foraged from wild habitats, including forests and open spaces (roadsides and wastelands). We confirmed the taxonomic identification for sixteen mushrooms. The mushrooms identified belonged to mycorrhizal, termitophilic and saprobic species. Nutritive values were assessed through laboratory analysis (n=8) and secondary review (n=3). Most mushrooms contained significant amounts of proteins (*Amanita hemibapha, Russula delica*, and *Termitomyces heimii*), iron and total dietary fibre (*Termitomyces* and *Astraeus*). Household consumption patterns revealed once-to-twice-a-week household consumption (30-60%). Thus, identifying approaches to increase consumption, like strategies for local propagation and education about wild mushrooms, could help in leveraging their nutritional potential.

## Abbreviations

FAO
: Food and Agriculture Organization
FFQ
: Food Frequency Questionniare
FGD
: Focus Group Discussion
HH
: Household
NABL
: National Accreditation Board for Testing and Calibration Laboratories
PPS
: Probability Proportional to Size Sampling
RDA
: Recommended Dietary Allowance
ST
: Scheduled Tribe
TEK
: Traditional Ecological Knowledge

## Introduction

Among the strategies explored to sustainably feed the world by 2050 and enhance nutrition security are approaches to maximize productivity and leverage local biodiversity to ensure the long-term sustainability of food systems. Promoting underutilised foods has been recognized as a feasible approach, calling them the “Future Smart Food” .^1^ The Food and Agriculture Organization (FAO), acknowledges the role of wild mushrooms in nutrition and livelihood maintenance ^2^ among marginal communities. It identifies mushrooms as one of the items that can help to preserve the foundations of our eroding food systems.^3^

Wild edible mushrooms are often referred to as superfoods and can improve dietary diversity and quality. Over 2100 species have been reported to be edible from an existing species range estimated between 50,000 and 100,000, of which the highest number of wild edible mushroom species are reported from Asia.^4^ These have traditionally been ascribed great economic and cultural importance among communities for providing food and medicine.^5^ Traditional ecological knowledge (TEK) regarding these species is passed on to generations, largely orally by local communities.

Documentation of the nutritive and non-nutritive values of wild edible mushrooms has expanded in the last few decades, exploring their potential to meet nutritional requirements and medicinal value.^6^ Wild mushrooms have low lipid content, are rich reservoirs of proteins with excellent amino acid profiles, oligo and polysaccharide content, minerals and vitamins.^7–10^

Wild mushrooms are found across India, from the Himalayan region to the Western Ghats and the Northeast. India has a very mature ethnomycological community with extensive morphotaxonomical and phylogenetic documentation on regional wild mushrooms.^11^ New species are continually being discovered as access to hitherto remote biodiverse areas becomes easier.^12–19^

The indigenous populations of India, described formally as scheduled tribes (ST), span across many states, with the ST population in Jharkhand comprising 26.2% of the total state population with over 32 indigenous communities.^20^ These indigenous communities mainly consist of subsistence farmers and small landholders. These marginalised communities are often undernourished, with health and nutritional indices lagging behind the general population.^21–23^ Our studies in this region have found many indigenous, nutrient-rich foods from local biodiverse environments and a deep, cultural repository of TEK.^24^ Among them were dozens of wild edible mushroom species observed to be regularly foraged. This information is pertinent since mushroom species are particular to climactic and geographic regions.

A few studies have analysed the role of wild edible plants and their potential for meeting the food and nutrition requirements and supplementing household (HH) incomes of indigenous communities.^8,13,18,25–27^ However, composite information on available species, nutritive values and consumption patterns is generally sparse, specifically from a biodiversity-rich region like Jharkhand. The discovery and documentation of wild mushrooms with cultivable potential through continuing research can significantly augment the production and consumption of these diverse nutrient-rich resources, boosting nutrition quality while providing a means of livelihood.^2,28^ As we observed regular seasonal consumption and availability of mushrooms among the indigenous communities, we explored the types of mushrooms available in four indigenous communities of Jharkhand, along with information on their consumption patterns, preferences, and determinants of consumption. We have also documented their nutritive values through analysis as a part of our study and obtained them from published literature. Thus, this paper synthesizes the findings on nutrient composition and consumption of commonly consumed mushrooms across Sauria Paharia, Santhal, Munda and Ho communities of Jharkhand, India.

## Materials and Methods

### Study Locale

This study was conducted in three selected districts of Jharkhand: Godda in the north-eastern part of the state (for Sauria Paharia and Santhal communities), Khunti (for Munda community) and West Singhbhum (for Ho community), in the southern part of the state (Supplementary Figure 1). All three districts have a wide range of forest cover and a rich biodiversity.^29–31^ These districts were chosen because they have a large proportion of the indigenous communities of interest.

### Study Design and Study Duration

This cross-sectional mixed-methods study was conducted using qualitative and quantitative enquiries to document the availability, taxonomic classification, nutritive values, and consumption frequency of wild mushrooms among selected indigenous communities in Jharkhand. This work was part of a larger project with an overarching objective of exploring the indigenous food systems of tribal communities of Jharkhand and understanding their impact on the nutritional status of tribal women and children.^32^ The data collection was conducted between March 2018 and February 2022.

### Sampling Framework and Study Population

A 2-stage cluster sampling design was followed in all four communities. In the first stage, administrative blocks were purposively chosen based on their accessibility and geographical distribution. Using a tribal village list from Census 2011,^20^ villages from each of the selected blocks were randomly selected using probability proportional to size (PPS) sampling. A detailed list of selected blocks and villages in each district is provided in Supplementary Table 1. In the second stage, all selected villages were visited in each tribal community, and a house-listing exercise was done to construct the sampling frame of all eligible HHs (i.e., presence of 1 non-pregnant woman in reproductive age group and one child (6–54 mo in a HH). In the case of >1 eligible woman in an HH during the house listing, one woman was randomly selected for the interview using the Kish table.^33^ The qualitative enquiries were conducted in randomly selected villages till the point of theoretical saturation was achieved. The study respondents included adult men, women, and elders, who were identified using snowball sampling. The quantitative enquiries were conducted in eligible HHs.

### Study Procedures

**Fig. 1:**
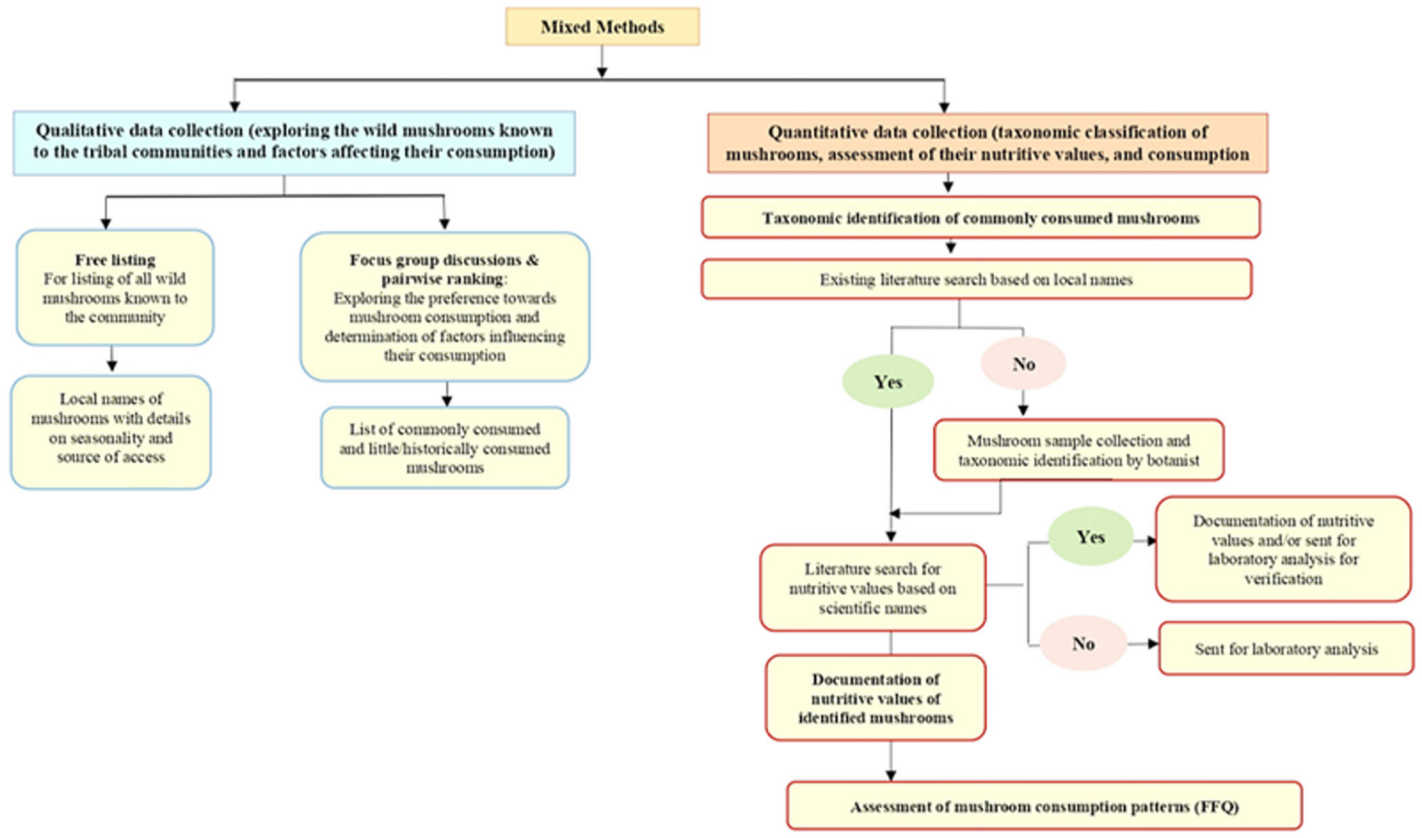
Detailed flow chart on the methodological approach used in the study

Qualitative and quantitative data collection were conducted to document the availability, taxonomic classification, nutritive values, and consumption frequency of wild mushrooms among selected indigenous communities in Jharkhand. A detailed flow chart on the methodological approach used in the study is provided in Figure 1.

### Qualitative Methods

#### Focus Group Discussions

Qualitative enquiries were conducted using focus group discussions (FGDs), which elicited information on the community's availability, access, and utilization of indigenous foods. A total of 35 FGDs (Sauria Paharia (n=11), Santhal (n= 8) Munda (n=9), Ho (n=7)) were conducted using pre-tested FGD guides that facilitated a free listing exercise to identify the range of foods including wild mushrooms consumed by the indigenous communities. In each FGD, the information was obtained from a group of 6-10 adult participants aged 18-65 years. The detailed FGD process and free listing are given in Supplementary Methods.

### Quantitative Methods

#### Mushroom Collection, Identification, and Nutritive Analysis

The taxonomic classification of mushrooms and assessment of their nutritive values were done for commonly consumed mushrooms in all four indigenous communities. For this purpose, a list of commonly consumed mushrooms, identified through qualitative enquiries, was prepared. Local names were used to search secondary literature for scientific names. If no information was available in secondary literature, then samples of mushroom varieties were collected for taxonomic identification from two field sites (Godda and West Singhbhum) based on their availability during the monsoon season. A botanist with extensive experience in the taxonomic classification of indigenous foods of Jharkhand guided the sample collection (Supplementary Box 1) and taxonomic identification. After taxonomic identification, the nutritive values of these identified mushrooms were searched in Indian food composition tables (IFCT) ^34^ and other peer-reviewed literature.^35–37^ If no information was available in secondary literature, the mushroom samples were collected from the field sites and sent for nutritive analysis to a National Accreditation Board for Testing and Calibration Laboratories (NABL) accredited laboratory. For certain varieties (like *Termitomyces* and Amanita species) for which nutritive values were available in published literature, laboratory analysis was done to verify their nutritive content. The collection for nutritive analysis was done as per a standard protocol developed as part of a larger study being conducted in these communities.^32^ The nutrient analysis was done at the NABL-accredited laboratory. The parameters analysed included energy, protein, carbohydrate, fat, dietary fiber, vitamin A (as beta-carotene), vitamin C, vitamin B1, vitamin B2, folate, iron, calcium, zinc, phosphorous, and vitamin D. The analyte values were reported per 100 g of edible weight (fresh mushroom sample). The details of the methods used for specific nutrients and the limit of quantification are provided in Supplementary Table 2.

#### Food Frequency Questionnaires

To assess the mushroom consumption patterns at the HH level, a food frequency questionnaire (FFQ) was administered by trained nutritionists in the monsoon season (Sauriya Paharia (n=120), Santhal (n=79), Munda (n=160), Ho (n=60)). This enquired about the frequency of consumption of different mushrooms within the HH over the past month. Commonly consumed mushrooms, identified through FGDs, were included in the FFQ, and their consumption frequencies were assessed through nine predefined categories ranging from “never” to “2 or more times per day”.

#### Data Analysis

All the FGDs and interviews were recorded and transcribed from local dialects (Paharia/Santhali/Mundari and Ho) to Hindi and then translated into English. The transcripts were used to generate a list of all the mushrooms known to the community. Further, based on the inputs from the participants during the FGDs, the listed mushrooms were categorized into commonly consumed and little consumed/historically consumed foods. Data from FFQ were summarized using frequencies and percentages.

## Results

The four indigenous communities (Sauria Paharia, Santhal, Munda and Ho) were found to be residing in villages surrounded by lush green forests and/or hills. These communities were found to be predominantly engaged in smallholder subsistence farming. They accessed foods from diverse natural sources, including farmlands, kitchen gardens, forests, open spaces (wastelands and roadsides) and water bodies (ponds, rivers, lakes). While all four communities practised settled agriculture at plain farmlands, Sauria Paharias were also found to be engaged in slash-and-burn farming on small patches of forest lands (known as Kurwa). Apart from natural food sources, the indigenous communities regularly procured food items from the local markets and Government food security programs.

### Wild Mushrooms Consumed by Indigenous Communities of Jharkhand

During the free listing, seventy-six (76) edible varieties of wild edible mushrooms were reported across the four indigenous communities. Seventeen varieties were common across some tribes, while the rest were unique to the individual tribes. Table 1 provides the local/vernacular names of wild mushroom varieties reported by each community. The local name used for mushrooms in these communities was ut/ud/chati/hosdu.

Transgenerational knowledge for identifying edible varieties of mushrooms was primarily orally transmitted. The maximum varieties were reported in Ho (n=34) and Sauria Paharia (n=33), followed by Munda (n=25) and Santhal (n=20). Of 75 listed wild mushrooms, 47% (n=35) were presently consumed across all four indigenous communities (Table 1). In Santhal, Munda and Ho communities, about 45-50% of the wild mushrooms were presently consumed. Among Sauria Paharias, only 27% (9 out of 34 varieties listed) were consumed. A total of 40 mushrooms were rarely consumed or historically consumed across the four tribes due to their limited availability in shared spaces and nearby forests.

The pairwise ranking results in the Sauria Paharia community revealed a clear preference towards the mushroom Telo Kuti, which was highly preferred for its palatability (Supplementary Figure 2). Other wild mushrooms preferred by the community for their availability as well as taste included Takna (*Termitomyces* heimii), Kero, Maango, Jambuaajo (*Termitomyces* fuliginosus) and Patangllo (Astraeus asiaticus and Astraeus odoratus). In the Ho community, Gitil ud (*Termitomyces* microcarpus) was found to be the most consumed mushroom during pairwise ranking, followed by Rotkeh (Astraeus asiaticus and Astraeus odoratus) and Gein mushroom (Lactarius rajmahalensis). Easy availability was the main reason cited for the frequent consumption. Among Santhals, nine commonly consumed varieties were reported, while the Munda community reported twelve varieties being widely consumed during the FGDs. A wild mushroom, namely Rugda (Astraeus asiaticus and Astraeus odoratus), was particularly valued for its taste and flavour and fetched high prices in the market. The commonly consumed varieties reported are given in Table 1.

All the mushroom varieties were available during the monsoon season. They were foraged and collected from wild habitats, including forests and open spaces (roadsides and wastelands). The mushrooms were also infrequently sold in Haats if the collected amounts exceeded the families’ consumption requirement. None of the species were cultivated by any of the communities.

### Culinary Practices

The mushrooms were mostly stir-fried. They were cooked in mustard oil with vegetables like onions and tomatoes and added spices to make a gravy-based dish. In Munda and Ho communities, the mushrooms were also cooked with other seasonal vegetables (like ivy gourd) and/or indigenous leafy vegetables to prepare mixed vegetable dishes.

### Storage for Non-Seasonal Use

Sun-drying and storing mushrooms for later use was also reported to be common across the four communities.

### Taxonomic Classification of wild mushrooms

**Fig. 2:**
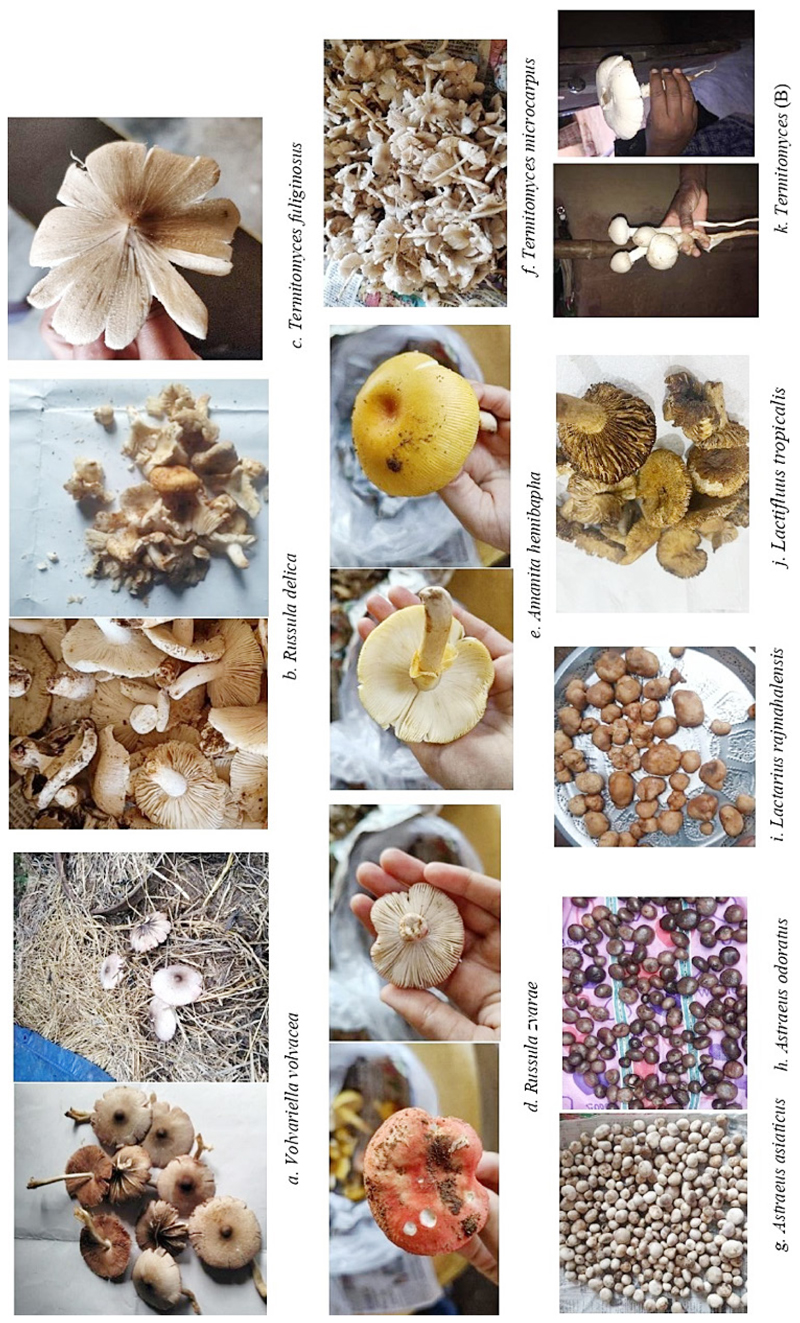
(a-k): Commonly consumed wild mushrooms of Jharkhand

Among the commonly consumed varieties of wild mushrooms (n=35), taxonomic identification was completed for sixteen mushrooms. The local and botanical names of the identified mushrooms are summarised in Table 2. The edible fungal species reported were chiefly from mycorrhizal, termitophilic and saprobic mushrooms. Among the identified mushrooms, *Russula* c.f kanadii, *Termitomyces* microcarpus, and *Termitomyces* heimii were the species reported to be most frequently consumed. Figure 2 (a-i) shows the pictures of all the species for which taxonomic identification was completed.

### Nutritive Values of Wild Mushrooms

Out of these identified species, we conducted a nutrient analysis of 8 wild mushroom species and varieties, and secondary data was available for three. Table 3 summarizes the nutrient composition of the mushrooms analyzed in the food testing laboratory, and Supplementary Table 3 describes the data extracted from published literature. Nutritive values could not be documented for mushrooms belonging to Lactifluus tropicalis (family Russulaceae), *Russula* densifolia, *Russula* zvarae and *Russula* psuedocyanoxantha.

The varieties Amanita hemibapha, *Russula* delica, and *Termitomyces* heimii were rich in protein, with content ranging from 10.5-39.02 g/100g, respectively. Similarly, from mushroom samples analysed at the food laboratory, varieties like Astraeus, Volvariella and *Russula* had high protein content (4.19-4.28 g/100g). Both the *Termitomyces* and Astraeus species analysed were seen to have high fiber content (>6 gm/100 g). *Russula* c.f kanadii, *Termitomyces*., and *Russula* delica had high iron (range: 6-20.1 mg/100g), and high calcium levels were reported in *Russula* delica (353.5 mg/100g) and the two varieties of Astraeus (185.6-193.4 mg/100 g). Substantial zinc content was reported in *Russula* delica (7.3 mg/100g) and *Russula kanadii* (3.5mg/100g) and both species of Astraeus (3.1-3.3 mg/100g). Among the vitamins, *Termitomyces*, Volvariella spp, and Astraeus odoratus had B1 levels/100g equivalent to RDA for adults. The vitamin D levels in mushrooms analysed at the laboratory (n=8) ranged between 2.2-2.6 µg/100g (approximately 150 IU) in mushrooms belonging to the genus *Termitomyces*

### Mushroom Consumption Pattern at Household Level

Using the FFQ for monsoon season, the wild mushrooms were seen to be consumed mostly once or twice a week in about 30-60% HHs in Sauria Paharia, Munda and Ho communities (Table 4). The frequency was reported to be highest among the Munda community, where at least 80% of the respondents reported consuming mushrooms at a frequency of 1-2 times a week (63%) or greater (20%). Majority of HHs in Sauria Paharia reported the consumption of varieties like Makko (53%), Parango (52%), and Maango (50%), while in Munda community, *Termitomyces microcarpus* and *Volvariella volvacea* were seen to be the commonly consumed varieties in 66% and 51% HHs respectively. In Ho community, *Russula c.f kanadii* was found to be the most consumed mushroom among 87% women. Santhals however reported lower weekly consumption (around 4%) and the reasons cited was delayed monsoon onset.

## Discussion

Our paper reports the local knowledge, availability, nutritive values, and consumption patterns of wild edible mushrooms among four indigenous communities of Jharkhand. Mushrooms can be a source of substantial protein and micronutrients such as iron, selenium, potassium, and magnesium. Due to their ability to grow without additional agricultural inputs, mushroom consumption can be an important contributor to diets among economically weaker communities with limited resources. Also, since the input resources are minimal so these foods can be an environmentally sustainable means of providing nourishment to nutritionally vulnerable communities. There are hundreds of wild mushrooms available across a geographically vast and biodiverse region like India with a astounding range of species growing in the rich flora in the country. Our study found TEK about 76 edible mushroom species, information on which are orally transmitted across generations. This information needs to be documented for the purpose of conservation of traditional knowledge and protection of the habitat that supports this rich biodiversity to ensure sustainable use of this wild food resource. Further, since the knowledge about the edibility is based on local wisdom and folk taxonomy, it’s scientific documentation is very critical. This knowledge is pertinent for a nutritionally vulnerable population which is struggling to maintain its nutrition security while trying to survive in a rapidly changing food environment. Use of underutilized wild foods has also been a central theme of FAO’s efforts to maximize use of local indigenous food resources.^1,39^

Our communities live in diverse habitats, including hilly terrains and forested grasslands. Thus, a range of mushroom genera were observed to be available such as Russula, *Termitomyces*, Lactifluus, and a macrofungus genera, Volvariella. The majority of species reported belonged to the genus Russula, of which nearly 180 taxa have been reported from India. Some of these were different from those reported in other regions such as the Himalayas and Western Ghats.^12,17,19^ So, our study is likely to inform the agricultural extension community regarding strategies for the suitability of species for local propagation. In parallel, species that are common with other habitats such as Volvariella volvacea, (the paddy straw mushroom) also provide an opportunity to attempt established cultivation practices in this region of India.^40,41^

Although these edible fungi are a valuable and cherished food item, local preferences between the communities in our study were somewhat different, possibly due to taste preferences as well as availability. Among the many wild mushrooms reported by the communities as commonly consumed, *Termitomyces microcarpus, Termitomyces heimii Amanita hemibapha, Volvariella volvacea* and *Russula c.f. alatoreticulata*, have been reported to be preferred for their taste among other populations.^42–44^ The frequency of consumption by the community was observed to be affected by the proximity of the villages to forests and accessibility for foraging, and the opportunity cost of collecting these mushrooms was one of the barriers to consumption.

Documentation of nutritive values of species reported being presently consumed among the communities revealed high amounts of protein, dietary fiber, iron, and zinc in many of these species. Other studies have also documented high nutritive values for these e.g., the *Astraeus spp*. and *Volvariella* were shown to have significant amounts of protein (>4 g/100 gm) and were also rich in vitamin B1, iron and zinc. The *Termitomyces* species had a high content of dietary fiber, vitamin B1, iron and zinc. The *Termitomyces* and Amanita species reportedly have high protein content, usually over 25% of their dry weight ^4,45,46^ and we found high protein content in *Termitomyces* heimii, consumed by our study communities. Similar observations on its protein content have also been documented in other studies.^25,47^ It has also been shown to have high concentrations of ergosterol, making *Termitomyces* heimii a rich source of Vitamin D precursor. Amino acid profile analysis of *Termitomyces* microcarpus and Amanita hemibapha species in other studies has shown high levels of total and essential amino acids (with T. microcarpus reported as having 13,106.2 mg/100 g).^10^

*Russula kanadii* had substantial protein content and was found to be particularly rich in iron and zinc content, containing these in concentrations equivalent to 100% of RDA. The genus *Russula* is known as one of the most widely distributed ectomycorrhizal agaric group, with 200 species known in Africa alone and used for edible purpose.^48,49^ *R. zvarae*, a species consumed by our communities has been documented from regions in Europe like Italy and Slovenia ^50^ but we did not find a documentation in published literature on this species from India.

We also administered dietary recalls among the HHs (data not reported); amounts consumed were low, ranging from 20-85 grams with a median of 45 and therefore not substantial. However, dietary intake diversity was also low among the communities surveyed. Thus, despite low amounts of mushroom consumption, their nutrient dense profile may add substantial value to the dietary diversity and intake profile of these indigenous communities.

Wild mushrooms have been explored for feasibility of cultivation to provide dietary supplementation and livelihood generation. Combining knowledge on consumption and habitats can help in understanding the various requirements for their propagation and make this a viable option for improving the intake diversity of the undernourished indigenous communities.^51^ Examples from India include *Pleurotus eryngii* and the *Volvariella volvacea*.^52^ The latter is valued as a high-quality human food source in South-East Asia and in some regions of India and is one of the most widely cultivated mushrooms worldwide. This choice is based on its nutrient rich profile, its low cost of production and shorter duration of growth compared to other species. Further, it has short shelf life (3-4 days) and thus could be specifically promoted for local cultivation and consumption to enhance dietary quality in these communities.^53^ Since the methodology and process for its cultivation has been adopted in many states, the propagation and use of this species, among others, can be a feasible strategy for augmenting dietary diversity and livelihood supplementation.^40,41^ So, domestication of this variety can be explored for our region given that community has knowledge and preference for this variety.

Climate variability could be affecting the availability and consumption of wild mushrooms in our region. The Santhal community reported delayed monsoon as one of the reasons for infrequent consumption observed during our dietary survey. Similar climatic effects have been reported on availability of wild mushrooms in Poland in a recent study on over 500 respondents.^54^ A study analyzing fungal response to climate showed that environmental changes due to global warming might lead to a drop in fungal yields.^55^ This would have an impact both on forest ecosystems and mushroom dependent economic activity.

Finally, as the nutrition transition makes inroads into the most remote communities, mushrooms can help offset the inevitable consequences of the nutrition transition like diabetes mellitus and cardiovascular disease. The presence of natural compounds such as fibers, polysaccharides, phenolics and alkaloids are known to have antidiabetic, antioxidant and antihyperlipidemic effects; these polysaccharides have been shown to act as prebiotics and modulate the composition of gut microflora, thus contributing to reducing insulin resistance among other beneficial metabolic effects.^56–58^

## Conclusion

In the present study, we found over 75 mushrooms on which the community had TEK, and over 35 were reported to be popular and cherished food items (e.g., *Termitomyces* and Astraeus spp.)among the diverse Indigenous communities of Jharkhand. These were consumed regularly when in season. We also identified and analysed the nutrient composition of sixteen commonly consumed and available mushroom species. They were nutrient-rich with a substantial content of protein, fiber, vitamins, and minerals and thus have the potential to enhance dietary diversity and quality, particularly among these undernourished communities.

Future work can extend the documentation of information about mushrooms among the various indigenous communities. Comprehensive data on species can help identify priority species for research and development both in agroforestry systems and during biodiversity conservation programmes. The species preferred by the communities can be included in the scope of government agricultural extension programmes to explore those amenable to propagation and farming, as has been done for the straw mushroom. Dissemination of information about their nutritional benefits can increase consumption. Locally feasible strategies could help propagate selected species to improve the diets of Indigenous populations and preserve knowledge of their nutritional and non-nutritional benefits.

## Supplementary Material

Supplementary Materials

## Figures and Tables

**Table 1:** Edible indigenous mushrooms consumed by Sauria Paharia, Santhal, Munda and Ho communities of Jharkhand, India

Sauria Paharia (n=34)	Santhals (n=20)	Munda (n=25)	Ho (n=34)
**Common varieties across all four tribes (n=17)**			
Pitodi	-	Rampatka/Patka ud	Sosoye ud
Balo	Sasag Tormil ut	-	Saga ud
-	Mutam ut	-	Idir ud/Bor ud
Chaandi ud	-	Badhayiud/Badheud	Muroom ud/Hende Ud
-	Kud ut	Koodeud	Koode ud
Mokron	Ootach ut	Patda ud	Pata ud
Chaariyoni	Muchi/mushi ut	Gitilud	Gitil ud
Aero Hosdu	Sim ut	Simoodali/simdaliud	Simdali ud/Rang
			biranga Pata ud
Hedro	-	Tormodaud/Bhorondaud	Sasang ud
Jambuajo	Tormil ut	Lechre ud /kundaud	Kunyad ud
Takna	-	Marangud/Bunum ud	Bunum ud
Gobri Hosdu	Busub ut	Piri ud	Pual chattu/Busub
-	Tumba ut	Tumbaud	Tumbe ud
-	-	Indi ud	Indi ud
-	-	Haathikata ud	Hathi Manda Ud
Patangallo/Phutka	Putka/Puttu chati	Rugra/Putuh	Potkeh/Rotkeh/ Rugda
Ganda budi		Endeud/Gendeud	Gein
**Unique varieties across the 4 tribes (n=58)**			
Kero/Orho/Korho	Butu	Koyaansakam	Angar Ud
Telokuti/Telokuto	Dhaamundi chati/Damand	Aataud	Eede Ud
Adro/edro/endro	Sem/semu chati	Kurthiud	Ude Ud
Maangro/Maako	Jangali chati	Chokerotte	Aatta Ud/Adaa Ud
Tero	Duma chati	Bengputu	Paiteh ud
Parango	Durga chati	Gomaud	Chimboor
Baado	Ortoot chati	Bedaud	Loyong Ud
Naango	Tugi chati	Dasayeud	Uju ud
Baansosu	Mandbhaat chati	Lundiud	Kadaye/Kadhai ud
Kaijo	Dhadwa	Chandna Rugra/ Chandna Putuh	Patka ud
Baanipoto			Cheeru ud
Baalco			Hashangar ud
Ado			Bair poga
Kerusudo			Surjum ud/Sal poga
Kuttapuda			Neem poga
Bandho aero			Aam poga
Bagdoto			Daru poga ud
Jhinganu/Jhingan			
Nalo Osu			
Patla aero			
Isuno			
Jinpro aero			

Note: Mushrooms in bold were found to be commonly consumed across the four indigenous communities

**Table 2:** Taxonomic classification of some frequently consumed indigenous mushrooms in Jharkhand (n=16)

S.no.	Scientific Name	Sauria Paharia Name	Santhal Name	Munda Name	Ho Name
1	*Russula delica* Fr.	*Pitodi*	NA	*Rampatka/Patka ud*	*Sosoye ud*
2	*Lactifluus tropicalis* A. Ghosh, I. Bera, D. Chakr. & Hembrom	*Balo*	*Sasag ut*	NA	*Saga ud*
3	*Termitomyces* (A)	NA	*Mutam ut*	NA	*Idir ud/Bor Ud*
4	*Russula densifolia* Seer, ex Gillet	*Chandi ud*	NA	*Badhayiud/Badheud*	*Muroom ud/Hende ud*
5	*Russula psuedocyanoxantha* Paloi, K. Acharya & S. Khatua	NA	*Kud ut*	*Koodeud*	*Koode ud*
6	*Russula c.f kanadii*A.K. Dutta & K. Acharya	*Mokron*	*Ootach ut*	*Patda ud*	*Pata ud*
7	*Termitomyces microcarpus* (Berk. & Broome) R. Heim	*Chaariyoni*	*Muchi/mushi ut*	*Gitilud*	*Gitil ud*
8	*Russula zvarae* Velen.	*Aero Hosdu*	*Sim ut*	*Simoodali/simdaliud*	*Simdali ud/Rang Birange* *Pata Ud*
9	*Amanita hemibapha* (Berk & Broome) Sacc.	*Hedro*	*Putka/Puttu chati*	*Tormodaud/Bhorondaud*	*Sasang ud*
10	*Termitomyces fuliginosus* R. Heim	*Jambuaajo*	*Tormil ut*	*Lechre ud /kundaud*	*Kunyad ud*
11	*Termitomyces heimii* Natarajan	*Takna*	NA	*Marangud/Bunum ud*	*Bunum ud*
12	*Volvariella volvacea* (Bull.) Singer	*Gobri Hosdu*	*Busub ut*	*Piri ud*	*Puaal Chattu*
13	*Astraeus asiaticus*^1^ Phosri, M.P. Martin & Watling ^1^	*Patangallo/Phutka*	*Putka/Puttu chati*	*Rugra/ Putuh*	*Potkeh/Rotkeh/ Rugda*
14	*Astraeus odoratus*^1^ Phosri, Watling, M.P. Martin & Whalley			*Chandna Rugra/* *Chandna Putuh*	
15	*Lactarius rajmahalensis Hembrom,* *K. Das & A. Parihar*	*Ganda budi*		*Endeud/Gendeud*	*Gein*
16	*Termitomyces* (B)	*-*	*-*	*Indi ud*	*Indi ud*

Mushroom identified based on the local name from secondary literature: ^138^

**Table 3:** Nutritive values of wild mushrooms analyzed in the laboratory

s. No.	Scientific name	Local names	Energy (Kcal/ 100g)	Protein (g/ 100g)	Carbohydrate (g/ 100g)	Fat (g/ 100g)	Dietary fibre (g/ 100g)	β- Carotene (μg/ 100g)	VitC (mg/ 100g)	Vit B1 (mg/ 100g)	Vit B2 (mg/ 100g)	Total folate (μg/ 100g)	Iron (mg/ 100g)	Zinc (mg/ 100g)	Calcium (mg/ 100g)	Phosphorus (mg/ 100g)	Vit D (μg/ 100g)
1	*Termitomyces fuliginosus* R. Heim	*Jambuaajo/ Tormil ut/ Lechre ud /Kun -daud/Kunyad ud*	41.5	2.5	7.5	0.2	6.1	9.4	<1.25	1.7	0.5	2.9	6.6	0.9	11.2	9.9	2.2
2	*Russula c.f kanadii* A.K. Dutta & K. Acharya	*Mokron/ Ootach ut/* *Rata ud/ Patda ud*	28.84	4.19	3.02	<0.1	1.2	<200	<1.25	<0.2	<0.2	1.9	12.1	3.5	39.3	4.1	<0.01
3	*Termitomyces microcarpus* (Berk. & Broome) R. Heim	*Chaariyoni/ Muchi /mushi ut/ Gitil ud/ Gitilud*	66.76	3.52	13.2	<0.1	6.9	9.3	<1.25	0.2	0.2	2.9	10.8	0.6	9.6	24.3	2.6
4	*Volvariella volvacea* (Bull.) Singer	*Gobri Hosdu/* *Busuk ut/ Puaal* *Chattu/ Piri ud*	34.68	4.27	4.4	<0.1	1.7	<200	1.4	2.9	0.8	0.3	3.7	1.7	34.9	192.1	<0.01
5	*Astraeus odoratus* Phosri, Watling, M.P. Martin & Whalley	*Rugra* (black)	138	4.8	29.5	0.06	7.3	<0.01	<1.25	0.6	0.4	0.3	6.8	3.1	193.4	30.2	<5
6	*Astraeus asiaticus* Phosri, M.P. Martin & Watling	*Rugra* (white)	141	4.3	30.9	0.02	7.6	<0.01	<1.25	1.9	0.1	0.3	3	3.3	185.6	8.3	<5
7	*Termitomyces* (B)	*Indi ud*	38.35	2.2	6.9	0.2	4.9	<5.0	<1.25	1.5	0.3	5.2	4.1	0.4	4.9	17.9	1.6
8	*Lactarius rajma -halensis* Hembrom, K. Das & A. Parihar	*Gein*	77.1	2.7	16.6	<0.1	2.4	<200	<1.25	2.2	0.6	1.9	6.5	4.1	18.5	3.2	<0.01

Note: Analyte values reported per 100g of edible weight (fresh sample)

**Table 4:** Frequency of mushroom consumption at household level among indigenous communities of Jharkhand

Frequency of consumption	n (%)			
	Sauria Paharia (n=120)	Santhal (n=79)	Munda (n=160)	Ho (n=60)
Every day (1-3 times)	1 ((0.83%)		1(0.62%)	3 (5%)
5-6 days a week	6 (5%)		4(2.5%)	3 (5%)
3-4 days a week	3 (2.5%)		30(18.75%)	5 (8.3%)
1-2 days a week	40 (33.33%)	3(3.79%)	101(63.12%)	14 (23.3%)
Once in a fortnight	14 (11.66%)		7(4.37%)	27 (45%)
Once in a month	40(33.33%)	3(3.79%)	8(5%)	5 (8.3%)
Never	16(13.33 %)	73(92.40%)	9(5.62%)	3(5%)

## Data Availability

The manuscript incorporates all datasets produced throughout this research study.
