# Supplementary material for: Wild Edible Mushrooms of Jharkhand: Nutrient-Dense Seasonal Foods to Improve Dietary Diversity among Indigenous Communities
Source: Curr Res Nutr Food Sci. Author manuscript; Available in PMC 2025 Apr 21. (PMC7617593; doi:10.12944/CRNFSJ.13.1.4)
Supplement: Supplementary Materials [file EMS204371-supplement-Supplementary_Materials.pdf]

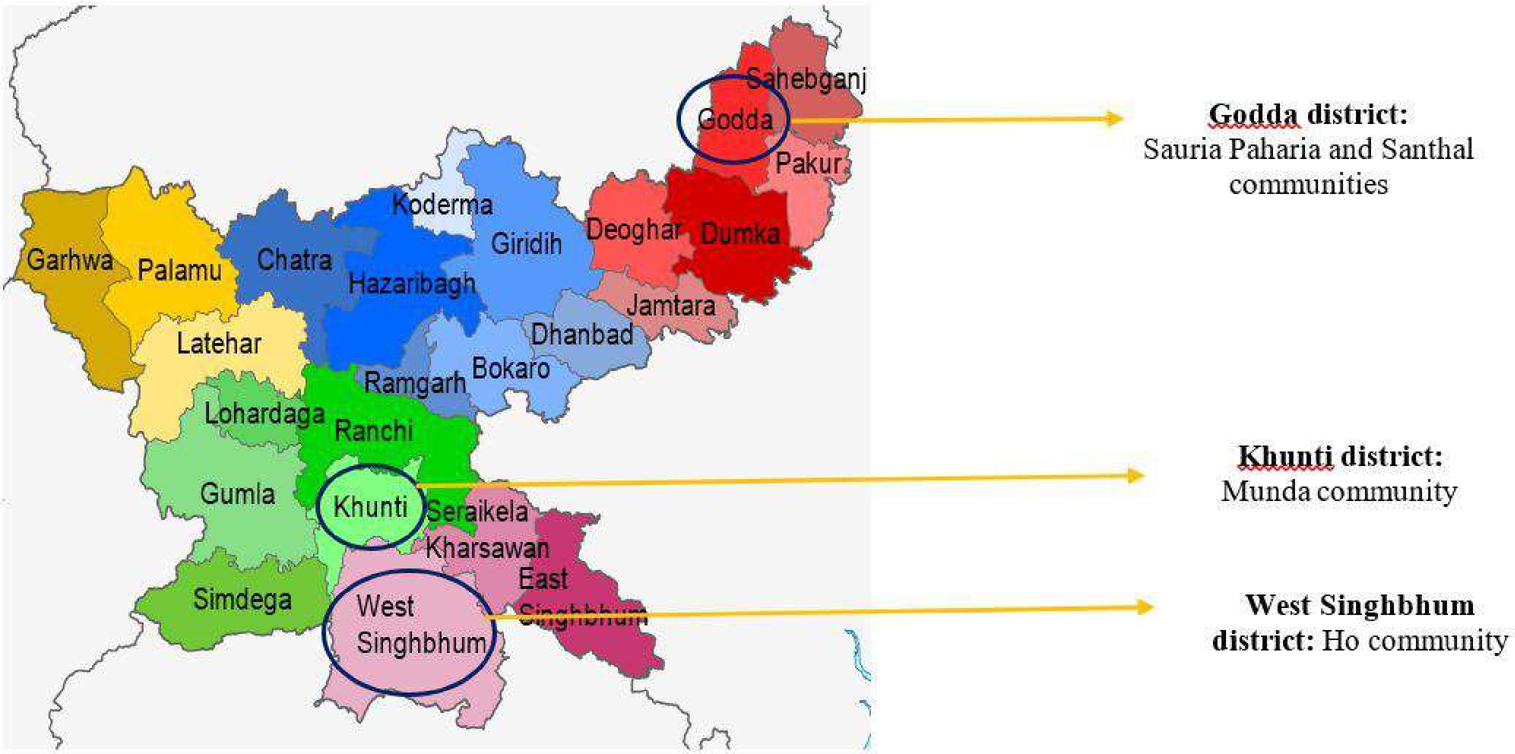

Supplementary Figure 1: Selection of study districts in Jharkhand, India

Supplementary Table 1: List of selected villages in Godda, Khunti and West Singhbhum district of Jharkhand, India

Godda district (Sauria Paharia)	Godda district (Santhal)	Khunti district (Munda)	West Singhbhum (Ho)
**Block 1: Sunderpahari**<br>1. Tasaria<br>2. Kusumghati<br>3. Paharpur<br>4. Chewo<br>5. Longodih<br>6. Nadgoda<br>7. Kheribari<br>8. Dahubera<br>9. Kusumaha	**Block 1: Sunderpahari**<br>1. Kaudhab<br>2. Bara Dhamni	**Block 1: Murhu**<br>1. Kudapurti<br>2. Gangina<br>3. Burju<br>4. Charid<br>5. Kurki<br>6. Siangkel	**Block 1: Khuntpani**<br>1. Loharda<br>2. Basakuti<br>3. Keadchelam<br>4. Baripi<br>5. Horlor
**Block 2: Boarijor**<br>10. Teletok<br>11. Kusumghati<br>12. Lutibahiar<br>13. Bara-amra<br>14. Kortica<br>15. Adro<br>16. Lohatamba<br>17. Chota Dumarhir<br>18. Rajapokhar	**Block 2: Boarijor**<br>3. Baghmara<br>4. Bara Boarijor<br>5. Sijua<br>6. Chitar Koti<br>7. Dhankoda<br>8. Amjhor	**Block 2: Torpa**<br>9. Urikel<br>10. Tati<br>11. Nichitpur<br>12. Jibolong	**Block 2: Chakradharpur**<br>6. Komae<br>7. Toklo<br>8. Hatnatodong<br>9. Bankitapi
	**Block 3: Poreyahat**<br>9. Bhatondha<br>10. Agiabandh<br>11. Kairasol<br>12. Dhobai<br>13. Dhobarni		**Block 3: Sonua**<br>10. Punipada
	**Block 4: Pathargama**<br>14. Pipra<br>15. Dighi<br>16. Patharkanimal<br>17. Khariani		

Supplementary Methods:

Description of Focus Group Discussion

Women were especially included in the discussions, as they were actively responsible for food collection and preparation. The FGDs were held in accessible areas such as Anganwadi centres (community-based centres for women and children) or in open areas in the villages. Participants were asked to list the food varieties, one by one, among each food group, including mushroom varieties. This was followed by eliciting information on the seasonality and habitat of each specific food item. After developing a free list of all the mushrooms known to the community, participants were further probed in order to develop the list of commonly consumed mushrooms vs. those that were little used or historically consumed. The perceptions on the reason why specific mushrooms were preferred or little used (e.g., availability, access, and/or taste) were also captured. In Sauria Paharia and Ho community, pairwise ranking method (Narayanasamy 2009) was used to assess preferences for specific mushrooms over others. For this, after the free listing exercise, the participants were asked to identify 4–5 preferred mushrooms, with preferences based on criteria of taste and availability. These preferred foods items were then tabulated as a matrix on a flip chart.

Supplementary Box 1: Standard guidelines followed for collection and storage of wild mushrooms

1. The habitats of mushrooms were identified.
2. Once identified, details like local names of the mushroom, its location (village name), GPS coordinates (i.e., latitude and longitude) and the date of sample collection, were captured in a form.
3. After recording of the details, photographs of the mushroom's gills, cap, stipe, base, rhizomorphs, upper and lower parts of the mushroom, were clicked
4. Approximately 250g of the mushrooms for each sample was collected for taxonomic identification. The samples were gently cleaned to remove any dirt or impurities if present.
5. The mushrooms were then placed in an airtight jar/ container, dipped in 70% ethyl alcohol. Each mushroom samples were placed in separate jars and labelled.
6. The labelled jars were given to botanists for taxonomic identification of the mushroom samples.

Supplementary Table 2: List of parameters and relevant methodological details for nutrient analysis

S. No	Test Parameter/Standard	Methodology	Reference method	Limit of Quantification (LOQ)
1.	Energy (Kcal/100gm)	Gravimetric method	IS:9487-1980(RA-2005)	1.0
2.	Protein (g/100gm)	Titrimetric method	FSSAI Lab Manual	0.1
3.	Total Fat (g/100gm)	Gravimetric method	IS:9487-1980(RA-2005)	0.02
4.	Total Carbohydrate (g/100gm)	By difference	IS:1656-2007	0.1
5.	Dietary Fibre (g/100gm)	Enzymatic Gravimetric method	AOAC 20th edition 985.29	0.5
6.	Vitamin A (as β- carotene) (µg/100gm)	High Performance Liquid Chromatography (HPLC)	QA.16.5.163	200
7.	Vitamin B_1,B_2 (mg/100gm)	High Performance Liquid Chromatography (HPLC)	QA.16.5.9	0.2
8.	Vitamin C (mg/100gm	High Performance Liquid Chromatography (HPLC)	IS:5838-1970 (RA-2005)	1.0
9.	Folic acid (µg /100gm)	High Performance Liquid Chromatography (HPLC)	QA.16.5.9	0.1
10.	Calcium, Iron, Zinc (mg/100gm)	Inductively Coupled Plasma Mass Spectrometry (ICP-MS)	QA.16.5.2/AOAC 20th edition	0.05
11.	Phosphorous (mg/100gm)	Spectrophotometric method	IS: 14828:2000	NA
12.	Vitamin D (µg/100gm)	High Performance Liquid Chromatography (HPLC)	QA.16.5.3	0.01

Sauria Paharia community

Rank	Indigenous mushrooms	Scores
**1**	*Takna*	5
**2**	*Tero*	4
**3**	*Maango*	3
**4**	*Kero*	2
**5**	*Balco*	1
**6**	*Ado*	0

Ho community

Rank	Indigenous mushrooms	Scores
**1**	*Gitil ud*	4
**2**	*Rotkeh ud*	3
**3**	*Gein*	2
**4**	*Pata ud*	1
**5**	*Idir ud*	0

Supplementary figure 2: Example of pairwise ranking and scores to indigenous mushrooms in Sauria Paharia and Ho community of Jharkhand, India

Supplementary Table 3: Nutritive value of mushrooms obtained from published literature

S. No.	Scientific name	Local name	Energy (Kcal/ 100g)	Protein (g/ 100g)	Carbohydrate (g/ 100g)	Fat (g/ 100g)	Dietary fibre (g/ 100g)	β- Carotene (µg/ 100g)	Vit C (mg/ 100g)	Vit B1 (mg/ 100g)	Vit B2 (mg/ 100g)	Total folate (µg/ 100g)	Iron (mg/ 100g)	Zinc (mg/ 100g)	Calcium (mg/ 100g)	Phosphorus (mg/ 100g)	Vit D (µg/ 100g)
**1.**	*Amanita hemibapha* (Berk & Broome) Sacc.	*Hedro/ Putka/Puttuchati/ Sasang ud/ Tormodaud/ Bhorondaud*	430	10.5	82.9	5.6	11.8	NA	NA	NA	NA	NA	5.9	0.4	8.4	515	NA
**2.**	*Russula delica* Fr.	*Pitodi/ Sosoye ud/ Rampatka/Patka ud*	289	39.02	35.6	7.9	20.1	NA	NA	NA	NA	NA	20.1	7.3	353.5	434.2	NA
**4.**	*Termitomyces heimii* Natarajan	*Takna/ Bunum ud/ Marangud/Bunum ud*	311	28.5	N/A	12.35	22.72	NA	NA	NA	NA	NA	0.1	NA	0.01	NA	NA

References: 1. Ravikrishnan et al., 2017; 2. Khatua et al., 2021 ; 3. Gunasekara et al., 2021
